# Oral Supplementation with Algal Sulphated Polysaccharide in Subjects with Inflammatory Skin Conditions: A Randomised Double-Blind Placebo-Controlled Trial and Baseline Dietary Differences

**DOI:** 10.3390/md21070379

**Published:** 2023-06-27

**Authors:** Lauren A. Roach, Barbara J. Meyer, J. Helen Fitton, Pia Winberg

**Affiliations:** 1 School of Medical, Indigenous and Health Sciences, Molecular Horizons, Illawarra Health and Medical Research Institute, University of Wollongong, Wollongong, NSW 2522, Australia; lroach@uow.edu.au; 2 RDadvisor, Hobart, TAS 7006, Australia; drfitton@rdadvisor.com; 3 Venus Shell Systems Pty Ltd., Nowra, NSW 2540, Australia; pia@venusshellsystems.com.au

**Keywords:** SXRG84, seaweed, skin, atopy, sulfated polysaccharide, anti-inflammatory, psoriasis, Palmoplantar Keratoderma

## Abstract

We examined the effect of a dietary seaweed extract—sulfated xylorhamnoglucuronan (SXRG84)—on individuals with inflammatory skin conditions. A subgroup analysis of a larger trial was undertaken, where 44 participants with skin conditions were enrolled in a double-blind placebo-controlled crossover design. Subjects ingested either SXRG84 extract (2 g/day) for six weeks and placebo for six weeks, or vice versa. At baseline, six- and twelve-weeks inflammatory markers and the gut microbiota were assessed, as well as skin assessments using the dermatology quality of life index (DQLI), psoriasis area severity index (PASI) and visual analogue scales (VAS). There were significant differences at weeks six and twelve for pro-inflammatory cytokines IFN-γ (*p* = 0.041), IL-1β (*p* = 0.030), TNF-α (*p* = 0.008) and the anti-inflammatory cytokine IL-10 (*p* = 0.026), determined by ANCOVA. These cytokines were all significantly higher at six weeks post placebo compared to twelve weeks post placebo followed by SXRG84 treatment. A total of 23% of participants reported skin improvements, as measured by VAS (mean difference 3.1, *p* = 0.0005) and the DQLI score (mean difference -2.0, *p* = 0.049), compared to the ‘non-responders’. Thus, the ingestion of SXRG84 for 6 weeks reduced inflammatory cytokines, and a subset of participants saw improvements.

## 1. Introduction

Microbiomes of both the skin and the gut are altered in skin disorders. Dietary modulation of systemic inflammation via the gut microbiome may be an effective tool in the management of atopic skin diseases [1].

Several skin conditions have been associated with a dysbiosis of the gut microbiota, such as psoriasis, eczema and rosacea [2]. Because of this, both probiotics and prebiotics have been identified as a potential means of addressing such conditions [3]. Probiotics have been successful in reducing symptoms of dermatitis [4] and reducing levels of inflammatory cytokines [5]. The effects are dependent on the species of bacteria, the dose, method of administration, duration and the environmental factors of the host [6]. In addition, prebiotics [7] such as konjac glucomannan resulted in a suppression of Immunoglobulin E, which resulted in the prevention of dermatitis development in a mouse model [8].

Sulfated xylorhamnoglucuronan rich extract (SXRG84) from Ulva sp. is a novel prebiotic seaweed extract with the potential to attenuate some of the symptoms of metabolic disorder, as described in our previous, larger study [9]. This larger trial looked at inflammation markers, metabolic markers and the microbiome in a crossover design. In the study described here, we undertook a subgroup analysis of the “Bio-Belly 2 trial” in which SXRG84 was ingested. The subgroup analysis undertaken here specifically identified subjects with inflammatory skin conditions.

The aim of this study was to investigate the effect of SXRG84 administration on symptoms of inflammatory skin conditions in a randomised, double-blind, placebo-controlled trial. It was hypothesised that improvements in inflammatory skin conditions would be observed after the ingestion of the novel seaweed extract SXRG84.

Furthermore, two case studies of skin conditions (Palmoplantar Keratoderma (PPK) [10,11,12] and Psoriasis—a common immune-mediated skin disease [13,14,15]—are presented as Supplementary Materials (Figures S1 and S2).

## 2. Results

### 2.1. Participants

**Figure 1:**
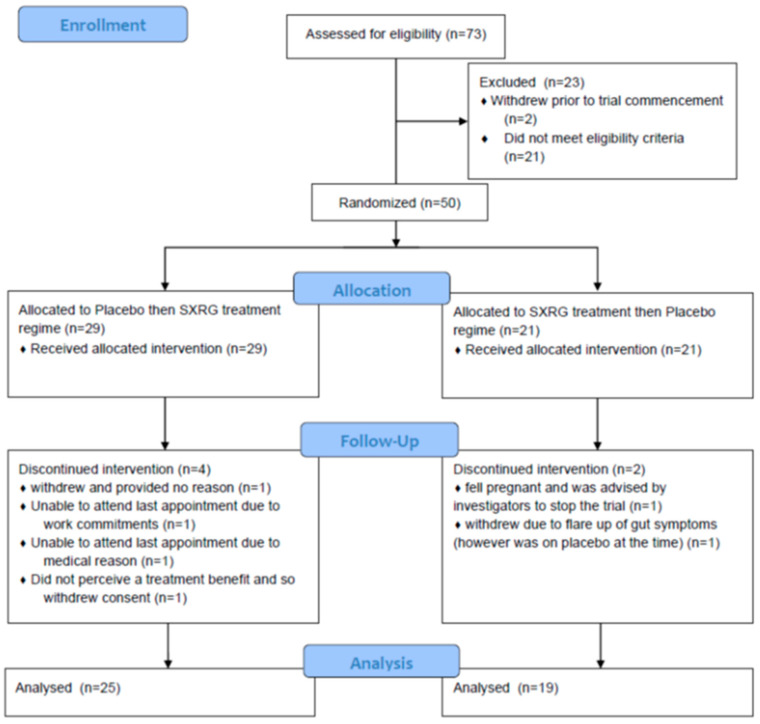
CONSORT flow diagram of participants with inflammatory skin conditions.

For the subset of participants with inflammatory skin conditions, a total of 50 participants were randomised to either regime. The reasons for participant withdrawal are summarized in Figure 1. A total of 25 participants and 19 participants were included in the analysis (group AB and BA, respectively).

### 2.2. Differences in Gut Microbiome between the Subsets of Skin Cohort and Non-Skin Cohort from the Original Clinical Trial

At baseline, a PERMANOVA determined that the cohort with inflammatory skin conditions used for this trial had a gut microbiome that differed in composition and abundance when compared to a cohort without inflammatory skin conditions (taken from the larger clinical trial [9] (*p* = 0.012 with log x+1 transformed data). A SIMPER analysis revealed the genera that contributed most to the dissimilarity between the two groups at baseline, and these are outlined in Table 1. A number of genera were also individually significantly different between the skin and non-skin cohorts at baseline, including *Bifidobacterium*, *Asteroleplasma*, *Butyrivibrio* and *Sarcina*.

### 2.3. Baseline Characteristics of Participants with Inflammatory Skin Conditions

There were no significant differences between the two regime groups at baseline for any of the outcome variables, except for BMI (Table 2). The regime group starting on the SXRG84 treatment and then crossing to the placebo treatment had a significantly higher BMI compared to the other regime group (*p* = 0.021).

### 2.4. Changes in Outcome Variables

When examining the changes in outcome variables at six weeks or twelve weeks, there were no significant changes for BMI, the skin measures (PASI, VAS and DQLI), CRP, IL-6 and IL-8. There were significant differences detected for IFNγ (*p* = 0.041), IL-1β (*p* = 0.030), TNFα (*p* = 0.008) and IL-10 (*p* = 0.026) between the four groups (Table 3). For IFNγ, group AA at six weeks post placebo was significantly higher than both group AB at twelve weeks post placebo then SXRG84 treatment (*p* = 0.009) and group BA at twelve weeks post SXRG84 treatment then placebo (*p* = 0.024). This pattern was also true for IL-1β, with group AA significantly higher at six weeks than both group AB (*p* = 0.050) and BA (*p* = 0.004) at twelve weeks. TNFα and IL-10 were also significantly higher in group AA at six weeks compared to group BA at twelve weeks (*p* = 0.001 and *p* = 0.003, respectively).

### 2.5. Changes in Gut Microbiota

To assess changes in the gut microbiota, a PERMANOVA was used to test for differences between the six and twelve week timepoints following either the placebo or SXRG84 treatment. There were no significant differences between gut microbiota composition and abundance for any timepoint (*p* > 0.05). A SIMPER analysis was then conducted to detect any common patterns in genus change that were evident after the SXRG84 treatment but not the placebo. There were three genera detected that increased in abundance after the SXRG84 treatment at six or twelve weeks and decreased in abundance after the placebo treatment; these were *Fusicatenibacter*, *Parabacteroides* and *Collinsella*.

### 2.6. Skin Responders and Non-Responders to the Intervention

Although there were no significant differences between the treatments and timepoints for the skin measures for the group as a whole, a subset of participants reported improvements in their skin condition. Ten participants reported noticeable improvements in their skin directly following the SXRG84 treatment (Table 4). An ANCOVA was then used to test this subset of ‘responders’ compared to the ‘non-responders’ while both on the active treatment (Table 5). The responder self-reported improvements were supported by a significant difference in the improvement of both the VAS (mean difference 3.1, *p* = 0.0005) and the DQLI score (mean difference −2.0, *p* = 0.049) and a trend towards an improvement in PASI (mean difference −1.6, *p* = 0.080) when compared to the non-responders. However, there were no significant differences between the responders and non-responders with regard to changes in inflammatory cytokines or CRP when tested with ANCOVA (*p* > 0.05). There were also no differences detected between the responders and non-responders in terms of baseline gut microbiota composition and abundance, or the changes in gut microbiota when assessed by PERMANOVA (*p* > 0.05 data not shown). Responders did not appear to have more severe skin conditions, as at baseline there were no significant differences between responders and non-responders with regard to PASI (*p* = 0.197), VAS (*p* = 0.678) and DQLI (*p* = 0.430).

However, significant correlations were detected between the change in PASI score (post - baseline) and the genera Actinomyces (*r* = 0.762, *p* = 0.017) in the responders. A change in the VAS was correlated with a change in the genera Kluyvera (*r* = −0.700, *p* = 0.036) in the responders. The change in DQLI score (post - baseline) was also significantly correlated with Butyrivibrio (*r* = −0.778, *p* = 0.014), Flavonifractor (*r* = 0.752, *p* = 0.019) and Sutterella (*r* = −0.846, *p* = 0.004) for the responders. At baseline, the skin measures were significantly correlated with a number of baseline genera normalised counts for the skin cohort as a whole (Table 6).

### 2.7. Dietary Intake

**Figure 2:**
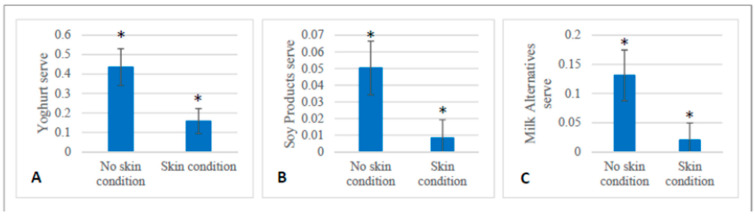
Dietary differences observed at baseline between participants with a skin condition (*n* = 42) and those without (*n* = 19). (**A**): Difference between the mean number of servings of yoghurt (±SEM) consumed at T1 (*p* = 0.02). (**B**): Difference between number of servings of soy products (±SEM) consumed at T1 (*p* = 0.03). (**C**): Difference between the mean numbers of servings of milk alternatives (±SEM) consumed at T1 (*p* = 0.04). * denotes a significant difference between groups *p* < 0.05.

**Figure 3:**
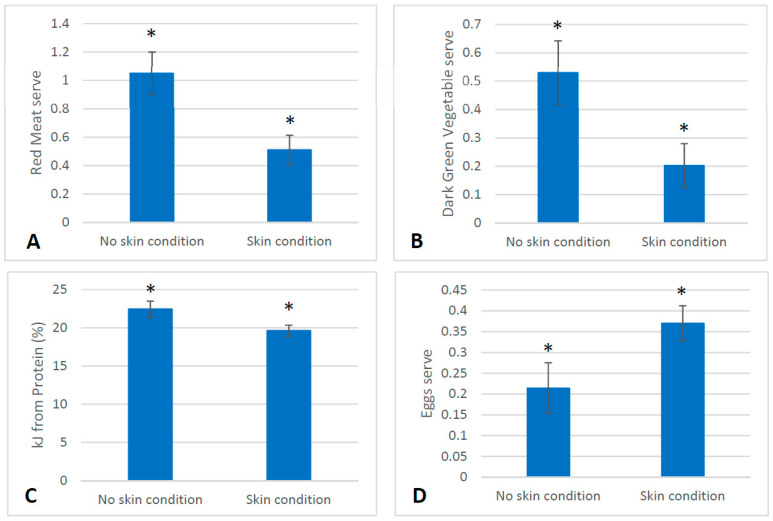
Differences in dietary intake observed at T2 between those with skin conditions (*n* = 42) and those without (*n* = 20). (**A**): Difference in mean servings of red meat (±SEM) consumed at T2 (*p* = 0.004). (**B**): Difference in mean servings of dark green vegetables (±SEM) consumed at T2 (*p* = 0.02). (**C**): Difference in mean percentage of kJ from protein sources (±SEM) at T2 (*p* = 0.03). (**D**)**:** Difference in mean servings of eggs consumed (±SEM) at T2 (*p* = 0.04). * denotes a significant difference between groups *p* < 0.05.

**Figure 4:**
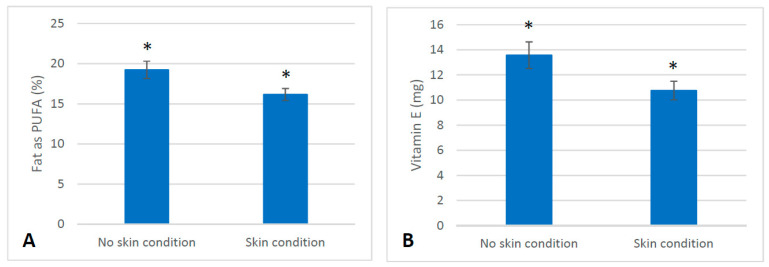
Differences in dietary intake observed at T3 between those with skin conditions (*n* = 41) and those without (*n* = 20). (**A**): Difference in mean intake of PUFA as a percentage of total fat intake (±SEM) at T3 (*p* = 0.02). (**B**): Difference in mean intake of Vitamin E (mg) (±SEM) at T3 (*p* = 0.03). * denotes a significant difference between groups *p* < 0.05.

At baseline, the dietary intake of servings of food differed between the skin cohort and the non-skin cohort from the larger clinical trial. At baseline, those with a skin condition consumed fewer servings of yoghurt, soy products and milk alternatives compared to the those without a skin condition (*p* < 0.05). (Figure 2). During the intervention, at six weeks (T2) those with a skin condition consumed less red meat and dark green vegetables and more eggs than those without a skin condition (*p* < 0.05) (Figure 3). At twelve weeks (T3), those with a skin condition consumed significantly less fat as polyunsaturated fatty acids (%) and vitamin E (*p* < 0.05) (Figure 4). All other servings of types of food and nutrients remained the same between the skin cohort and the non-skin cohort.

## 3. Discussion

This study investigated the effect of SXRG84 treatment on a range of inflammatory skin conditions. Although there were no significant skin improvements across the whole cohort, there were significant results for inflammatory cytokine reduction. A subset of participants experienced improvements in skin outcome measures. The microbiome assessment yielded a more complex picture.

Whilst the study was not powered for the number of consistent categories of skin conditions, it did indicate an improvement in 27% of thirty-eight psoriasis sufferers and both of two eczema sufferers, although not for the five other skin condition categories (*n* < 5 for all of them). Overall, 23% of participants reported significantly improved skin conditions following SXRG84 treatment across VAS (3.1, *p* = 0.0005) and DQLI (−2.0, *p* = 0.049), but with a non-significant trend of improvement in PASI (−1.6, *p* = 0.08).

Improvements in inflammatory cytokines were observed when subjects ingested SXRG84. INF-γ, IL-1β and TNF-α were all significantly higher six weeks post placebo vs. post SXRG84 treatment period. Reductions in cytokines could still be observed at twelve weeks after SXRG84 treatment which then crossed to placebo, suggesting a potential carryover or longer-term effect.

Improvements in cytokines have been reported previously with intervention trials for inflammatory skin conditions, particularly with probiotic treatment. These cytokine improvements can either be through the reduction of inflammatory cytokines, such as reductions in CRP, TNF-α and IL-6 in psoriasis patients after treatment with *Bifidobacterium infantis* [16], or through the upregulation of anti-inflammatory cytokines, such as increases in IL-10 shown in animal models in conjunction with skin improvements after probiotic treatment [17]. The role of prebiotics to impact on the inflammatory response is less clear in the literature, but this present study suggests that SXRG84 reduces pro-inflammatory cytokine levels in people with inflammatory skin conditions, even when this is not correlated with an improvement in symptoms.

At baseline, there was a difference in the gut microbiome between cohorts with and without skin conditions taken from the larger clinical trial [9]. A total of 28 taxa explained just over 90% of the difference between the groups (Table 1). The gut has been implicated in certain skin conditions previously, evidenced by children with atopic eczema having impaired mucosal gut barrier integrity compared to healthy controls, and psoriasis being more common in people with Crohn’s disease than those without [18]. Furthermore, a microbiomic signature has been proposed for people with psoriasis from the skin microbiome [19], and so it is possible that a gut microbiome signature for people with specific inflammatory skin conditions may also be established. Trials have reported differences in the gut microbiota between people with inflammatory skin conditions and those without, in particular an increase in Firmicutes and a depletion of Bacteroidetes in psoriasis patients. Additionally, the *Faecalibacterium* and *Blautia* genera were higher in psoriasis patients, while Bacteroides and *Paraprevotella* were higher in non-psoriasis controls [20]. In atopic dermatitis, the majority of trials investigated infants and have reported reductions in *Bifidobacterium* [21,22] *Staphylococcus aureus* [21] and mucin-degrading bacteria [23] when compared to healthy controls, while increases in lactic acid bacteria [22], *Escherichia coli* [24], *Clostridia* and a subspecies of *Faecalibacterium prausnitzii* [25] have been reported in infants with atopic dermatitis. In psoriasis, imbalances in the gut microbiota have also been reported with increases in certain pathogenic bacteria such as *Salmonella*, *E. coli*, *Helicobacter*, *Campylobacter*, *Mycobacterium* and *Alcaligenes* [26,27]. Decreases in *Bifidobacterium, Lactobacilli*, *F. prausnitzii*, *Parabacteroides*, *Coprobacillus* and *Coprococcus* have been shown in individuals with psoriasis [27]. These changes in the gut microbiota evident in skin pathologies are suggested to impair the ability of the gut to regulate immune responses, and lead to inflammation and skin conditions.

Here, we reported an increase in lactic-acid-producing bacteria *(Streptococcus)* and a reduction in mucin degrading bacteria *(Akkermansia)* in those with a skin condition. However, we found an increase in *Bifidobacteria* in those with the skin condition, in contrast to the published evidence described above. The fact that many of the changes we reported between those with and without the skin condition were not supported by published evidence may be because multiple skin conditions were included in this study that may differ amongst themselves with regard to gut microbiota. How individual skin conditions differ in gut microbiota composition is yet to be determined.

Furthermore, we detected differences in dietary intake between those with a skin condition and those without at baseline prior to any intervention treatment. Of note, those presenting with an inflammatory skin condition consumed fewer servings of yoghurt than those without a skin condition. Yoghurt, a fermented dairy product, contains probiotic bacteria, particularly lactic acid bacteria [28]. Yoghurt in itself has been beneficial for skin health in terms of moisture and elasticity when applied topically [29] and has improved the skin barrier function when ingested [30]. The ingestion of yoghurt in some individuals also improved symptoms of acne vulgaris [30] and atopic dermatitis [31], and as such is a noteworthy dietary difference between those with a skin condition and those without. In addition, differences in diet detected throughout the intervention collectively may suggest that those with skin conditions were consuming a less varied diet overall. Both PUFAs (particularly omega-3s) and Vitamin E were both decreased during the intervention period in the cohort with skin conditions, and these nutrients have also been implicated as beneficial for the inflammatory processes underpinning skin conditions such as psoriasis [32]

Three genera increased with SXRG84 treatment and decreased with placebo treatment, namely, *Fusicatenibacter*, *Parabacteroides* and *Collinsella.* These genera have generally been associated with positive health effects or positive health status, although some evidence is limited or conflicting. *Fusicatenibacter* has been shown to be higher in long term probiotic consumers (> 1 year) [33] and is associated with the consumption of Kefir [34]. *Fusicatenibacter* is reduced in people with adenocarcinomas who have received chemotherapy or radiotherapy when compared to healthy controls [35], and so increases in this genera are assumed to be positive. Certain species of *Parabacteroides* have shown specific health benefits and have been shown to respond to polysaccharide supplementation. Specifically, *Parabacteroides goldsteinii* respond to a high molecular weight polysaccharide fraction from *Hirsutella sinensis* (fungus) in high-fat-fed mice [36]. High-fat-fed mice supplemented with *P. goldsteinii* had less weight gain due to increased adipose tissue thermogenesis, improved insulin resistance, intestinal integrity and inflammatory levels (reduced serum IL-1β) [36]. Another species of *Parabacteroides*, *Parabacteroides distasonis*, also demonstrated anti-inflammatory properties by preventing an increase in pro-inflammatory cytokines such as IFNγ, IL-12, IL-17 and IL-16 in mice after induced acute or chronic colitis [37]. However, a more recent work contradicts this finding, with suggestions that *Parabacteroides distasonis* is both a prebiotic and a potential pathogen [38]. Lastly, *Collinsella* is a genera found to be decreased in people with irritable bowel disease compared to healthy controls [39]. However, once again evidence is conflicting as *Collinsella* has also been shown to correlate positively with circulating insulin and correlate negatively with dietary fibre intake in overweight and obese pregnant women [40]; this contradicts the findings presented here, where *Collinsella* increased after SXRG84. Although the evidence for these three genera are conflicting, they are all linked to inflammatory gastrointestinal conditions and as such it is plausible that they are connected to the anti-inflammatory effects that are reported here; however, direct causations cannot be determined.

There were no significant improvements in skin conditions for the group as a whole; however, a subset of participants did report improvements in their skin. It is unclear as to why a subset of the group responded to treatment and the remainder did not, as there were no apparent differences between the cytokine response or the gut microbiota response. The responders either had psoriasis (*n* = 8) or eczema (*n* = 2), and with only two eczema cases enrolled in the trial, further work would be valuable to determine effects in people with eczema. The dosage may be one reason for the difference in response; a case study report using the same SXRG84 extract was successful in improving symptoms of palmar plantar keratoderma with a 4 g/day dose for six weeks, while 2 g/day for six weeks was effective in a psoriasis case (see Supplementary File). However, when the subject from case report #1 was enrolled in the above clinical trial, there was no observed improvement after the 2 g/day dose of SXRG84, although her fasting blood glucose improved to normal levels (as the case study improvements were observed after a 4 g/day dosage). This would suggest that a 2 g/day dose is insufficient, and further work is needed to determine the optimal doses for various skin conditions.

To our knowledge, these are the first case reports for dietary seaweed fibre ameliorating symptoms of psoriasis (see Supplementary File). Improvements in psoriasis have been linked to other dietary interventions, such as energy restrictions with enriched omega-3 long chain polyunsaturated fatty acids [41] and gluten free diets [42].

Our approach in including multiple skin conditions may have complicated the results by including different conditions that also differed in their presentation of symptoms and the underlying pathogenesis of the condition. Also, the severity of skin condition varied greatly among subjects. Therefore, different conditions and the severity of conditions may respond to the SXRG84 differently; our subset of ‘responders’ were not all from the same category of skin condition.

## 4. Materials and Methods

### 4.1. Ethics and Clinical Trial Registration

This trial was approved by the University of Wollongong Human Research Ethics Committee, approval no. 2017/101, and was registered with the Australian and New Zealand Clinical Trial Registry (ACTRN12617001010381). The research was conducted according to the guidelines of the Declaration of Helsinki [43].

### 4.2. Participants and Study Design

Participants were enrolled in a larger, randomized, double-blind placebo crossover trial, assessing the effect of a seaweed SXRG84 extract on metabolic and inflammatory markers (Bio-Belly 2 trial [9]) in overweight adults. This secondary analysis included a subset of participants that presented with inflammatory skin conditions including psoriasis, eczema or rosacea; participants who presented with an undiagnosed inflammatory skin condition were characterized in the general category of “dermatitis”. The majority of participants were overweight; however, participants of a healthy weight with inflammatory skin conditions were included. Participants were excluded if they had recently consumed antibiotics (within the previous 2 months). This subset was utilized to test the effectiveness of a 2 g daily dose of SXRG84 on symptoms of inflammatory skin conditions. Participants were randomly assigned by a computer-generated sequence to one of two regimes of the crossover trial, either consuming the placebo for six weeks and then taking the SXRG84 treatment for the following six weeks, or taking the SXRG84 treatment for the first six weeks and then the placebo treatment for the following six weeks. Treatment allocation was conducted by an independent person. There was no washout period between the first six weeks and the second six weeks, as there were no perceived effects from the placebo treatment.

### 4.3. Clinic Visits

Participants attended clinic visits at the Illawarra Health and Medical Research Institute (IHMRI), Wollongong, Australia, between August and December 2017. Visits occurred at baseline; at week six, at the end of the first treatment arm, which coincided with the commenced of the second treatment arm; and at week 12, following the completion of the second treatment arm. At each clinic visit, participants provided a fasted blood sample via venepuncture by qualified phlebotomists into 10mL ethylenediaminetetraacetate (EDTA) tubes. Participants also provided a faecal swab using a “Gut explorer kit” from uBiome, San Francisco, USA (www.ubiome.com, accessed 1 August 2017). At each clinic visit, participants’ skin was photographed and graded using a psoriasis severity index (PASI) [44] by the investigator (LAR). Participants also completed a dermatology life quality index (DLQI) [45] and a visual analogue scale (VAS) where they were asked to grade the severity of the appearance of their skin from “the worst the condition has ever looked” to “condition has completely cleared up” along a linear line. Participants were also provided with supplements at each clinic visit.

Details of the SXRG84 extract have been published previously [9]. The placebo contained milled brown rice, and both treatments contained seaweed pigment (< 1%) to ensure visual consistency. Participants were instructed to consume 5 capsules per day to achieve a total dose of 2 g of SXRG84 per day. Participants and researchers were blinded as to in which order they consumed the treatments.

### 4.4. Blood and Faecal Sample Analysis

Blood samples were analysed for inflammatory cytokines using an immunoassay high sensitivity Luminex Panel by Crux Biolab, Melbourne, Australia (https://cruxbiolab.com.au/, accessed on 1 August 2017). The cytokines analysed were IFNγ (interferon gamma), IL-6 (interleukin 6), TNFα (tumor necrosis factor alpha), IL-1β (interleukin 1 beta), IL-8 (interleukin 8) and IL-10 (interleukin 10). C-reactive protein (CRP) was measured on a Konelab autoanalyser (Konelab 20XT, Thermo Fisher Australia Pty Ltd, Scoresby, Australia) using an immunoturbidimetric assay (kit code 981934) according to manufacturer’s instructions and obtained from Thermo Fisher Australia Pty Ltd (Scoresby, Australia). Faecal samples were analysed by uBiome using 16S rRNA sequencing.

### 4.5. Dietary Intake

Participants’ dietary intake was assessed by three 24 h recalls at baseline, which included one weekend day and two weekdays of intake. These were entered into Foodworks nutrient analysis software (Version 8, Xyris Software, Pty Ltd. (Xyris Pty Ltd., Brisbane, Australia). The databases AusBrands 2017 and AusFoods 2017 were used to identify foods consumed. Dietary intake was compared between those with a skin condition and those without a skin condition (from the larger clinical trial [9]).

### 4.6. Statistics

Data were analysed with SPSS Statistics version 21 (IBM Corporation, Armonk, NY, USA). Researchers were blinded to the treatment allocation during data analysis. Data were tested for normality using the Shapiro–Wilk test, and data that were not normally distributed are presented as median (25th, 75th percentile). The two regime groups at baseline were compared using the Chi-Squared test for gender, and for the remaining variables a T-test or Wilcoxon signed-rank test for data that were not normally distributed. To test if there was a difference in the change in any outcome variables, an ANCOVA was used. The ANCOVA tested the difference in each outcome variable across four groups, and for each ANCOVA the baseline value of the outcome variable was used as a co-variate. The four groups are outlined in Table 7.

The PRIMER-E software package (Version No.6, Plymouth Marine Laboratories, Auckland, New Zealand) was used to analyse the gut microbiota data, which were analysed at the genus level. Permutational multivariate analysis of variance was used to test the differences between the skin cohort from this study compared to the non-skin cohort from the larger clinical trial to detect any differences at baseline. Permutational multivariate analysis of variance was also used to investigate differences between the three timepoints (baseline, six weeks and twelve weeks) for the two regime groups, creating a total of six groups.

Statistical significance was set as *p* < 0.05. A SIMPER analysis (Version No.6, Plymouth Marine Laboratories, Auckland, New Zealand) was used to test which genera contributed to the differences between groups. Severe outliers were assessed visually using multidimensional scaling plots and were removed from analysis.

Spearman correlations were conducted between the changes in skin measures (PASI, VAS and DQLI) and the changes in gut genera for the participants who were considered responders whilst on the SXRG84 treatment. Correlations were also conducted between skin measures at baseline and gut microbiota genera at baseline.

## 5. Conclusions

In conclusion, SXRG84 ingestion has the potential to improve a subset of inflammatory skin conditions, potentially through the reduction of pro-inflammatory cytokines. However, the effect was not seen across all participants, and further work is required to determine the optimal dose, the duration of the trial and the specific skin conditions that may receive a benefit from such treatment. Collectively, these results indicate that there are differences in the microbiome profiles of people with skin conditions (less yoghurt was consumed in those with a skin condition), and that inflammation markers and skin conditions can be improved in some cases following treatment with SXRG84. Tying these four variables of gut microbiome, yoghurt consumption, skin condition and inflammation status together into causal or response relationships requires a larger cohort analysis of consistent skin pathology. Two supplementary case studies indicate the potential benefits in those with PPK and psoriasis, and warrant further investigation.

## Figures and Tables

**Table 1:** Dissimilarities between skin cohort and non-skin cohort at baseline determined by SIMPER analysis.

Genus	Average Abundance in Those with Skin Condition *n* = 35	Average Abundance in Those without Skin Condition *n* = 18	Contribution to Dissimilarity %	Cumulative %
*Thalassospira*	6.00	5.72	5.61	5.61
*Bifidobacterium*	6.50 ^a^	3.77 ^b^	5.05	10.66
*Kluyvera*	4.72	2.50	4.96	15.62
*Akkermansia*	6.43	6.97	4.90	20.52
*Sutterella*	6.83	7.61	4.48	25.00
*Catenibacterium*	3.23	3.87	4.47	29.47
*Parasutterella*	4.97	6.41	4.44	33.91
*Prevotella*	5.04	2.55	4.33	38.25
*Phascolarctobacterium*	7.83	6.98	4.20	42.44
*Finegoldia*	4.16	2.70	4.04	46.48
*Peptoniphilus*	4.03	2.40	4.00	50.48
*Asteroleplasma*	1.60 ^a^	3.54 ^b^	3.93	54.41
*Corynebacterium*	3.70	2.82	3.86	58.27
*Megasphaera*	3.12	1.66	3.86	62.13
*Butyrivibrio*	5.44 ^a^	5.74 ^b^	3.22	65.35
*Megamonas*	1.47	1.64	3.01	68.37
*Klebsiella*	1.62	1.53	2.99	71.35
*Alloprevotella*	1.00	1.75	2.63	73.98
*Peptoclostridium*	6.63	5.94	2.57	76.55
*Intestinibacter*	7.91	8.33	1.78	78.33
*Sarcina*	9.40 ^a^	9.88 ^b^	1.72	80.05
*Lachnospira*	8.72	8.10	1.69	81.74
*Erysipelatoclostridium*	8.08	8.20	1.50	83.24
*Subdoligranulum*	9.31	9.95	1.48	84.72
*Alistipes*	9.65	10.37	1.47	86.19
*Streptococcus*	8.14	7.58	1.46	87.65
*Anaeroplasma*	0.72	0.57	1.29	88.93
*Fusicatenibacter*	9.59	9.71	1.15	90.08

Data presented as percentage abundance. Different letters denote a significant difference between the genera individually between skin and non-skin groups determined by a Wilcoxon signed-rank test, significance determined by *p* < 0.05.

**Table 2:** Baseline demographics by treatment regime groups.

	Group AB: Placebo Then SXRG Treatment (*n* = 25)	Group BA: SXRG Treatment Then Placebo (*n* = 19)	*p*-Value ^1^
Gender, F, (%)	13 (52%)	9 (47%)	0.76
Age (years)	51.2 ± 14.6	53.6 ± 11.4	0.55
BMI	27.5 (24.8, 30.4)	29.7 (28.1, 33.3)	0.02
Skin Category			
Psoriasis, n (%)	17 (68%)	13 (68%)	
Eczema, n (%)	1 (4%)	1 (5%)	
Rosacea, n (%)	1 (4%)	1 (5%)	
Dermatitis, n (%) *	2 (8%)	3 (16%)	
PPK, n (%)	0 (0%)	1 (5%)	
DSAP, n (%)	1 (4%)	0 (0%)	
PPP, n (%)	3 (12%)	0 (0%)	
Skin Measures			
PASI ^§^	2.4 (0.6, 4.4)	3.0 (1.6, 4.9)	0.18
VAS ^§^	5.0 (4.1, 6.2)	5.0 (5.0, 5.0)	0.90
DQLI ^§^	3.0 (2.0, 5.5)	5.0 (3.0, 11.0)	0.11
Inflammation			
C-reactive protein (mg/L) ^§^	0.0 (0.0, 3.2)	0.0 (0.0, 1.7)	0.78
IFN-gamma (pg/mL) ^§^	3.2 (1.8, 5.1)	3.5 (1.8, 5.3)	0.95
IL-1 beta (pg/mL) ^§^	18.0 (9.2, 26.9)	16.1 (10.1, 26.3)	0.88
IL-6 (pg/mL) ^§^	11.9 (8.0, 20.2)	12.3 (8.4, 18.1)	0.84
TNF-alpha (pg/mL) ^§^	7.7 (3.9, 12.0)	5.4 (3.1, 12.9)	0.84
IL-10 (pg/mL) ^§^	1.3 (0.7, 2.3)	1.0 (0.8, 2.5)	0.91
IL-8 (pg/mL) ^§^	5.6 (3.7, 10.4)	5.4 (3.5, 7.5)	0.88

Data are presented as number and % for gender, mean ± standard deviation or median (25th and 75th percentile). SXRG84, sulfated xylorhamnoglucuronan; BMI, body mass index; PPK, palmar plantar keratoderma; DSAP, disseminated superficial actinic porokeratosis; PPP, palmar plantar psoriasis; PASI, psoriasis area severity index; VAS, visual analogue scale; DQLI, dermatology quality of life index. * Those presenting with an undiagnosed inflammatory skin condition were classed generally as Dermatitis ^1^. *p*-value determined by Pearsons Chi-Squared test (gender), *t*-test or Wilcoxon signed-rank test (§).

**Table 3:** Changes from the baseline at 6 weeks and 12 weeks after placebo or SXRG84 treatment.

	AA 6 Weeks from Baseline (Placebo) *n* = 25	AB 12 Weeks from Baseline (Placebo Then Active) *n* = 25	BB 6 Weeks from Baseline (Active) *n* = 19	BA 12 Weeks from Baseline (Active Then Placebo) *n* = 19	*p*-Value *
BMI	0.0 (−0.3, 0.6)	0.0 (−0.4, 0.4)	0.3 (−0.3, 0.4)	0.0 (−0.9, 0.4)	0.520
Skin Scores					
PASI	−0.4 (−1.7, 0.0)	−0.8 (−2.1, 0.0)	−1.2 (−1.8, 0.0)	−0.6 (−2.5, 0.4)	0.664
VAS	0.0 (−2.0, 2.2)	0.2 (−2.0, 2.5)	1.2 (−0.4, 3.1)	0.0 (−1.5, 2.5)	0.828
DQLI	−1.0 (−2.0, 0.5)	0.0 (−2.0, 1.0)	−2.0 (−5.0, 0.0)	−2.0 (−4.0, 1.0)	0.694
Inflammation					
C-reactive protein (mg/L)	0.0 (0.0, 2.2)	0.0 (0.0, 0.8)	0.0 (0.0, 6.1)	0.0 (0.0, 0.1)	0.559
IFN-gamma (pg/mL)	0.2 (−0.7, 1.6) ^a^	−0.4 (−1.8, 0.7) ^b^	0.4 (−0.9, 1.5) ^ab^	−0.8 (−2.7, 0.1) ^b^	0.041
IL-1 beta (pg/mL)	−0.3 (−4.5, 8.3) ^a^	−0.5 (−8.5, 4.9) ^b^	0.8 (−7.2, 5.7) ^ab^	−5.4 (−11.3, 0.2) ^b^	0.030
IL-6 (pg/mL)	1.1 (−4.9, 7.4)	−0.7 (−4.9, 3.6)	1.3 (−2.9, 3.9)	−1.3 (−6.0, 3.0)	0.284
TNF-alpha (pg/mL)	1.7 (−1.1, 7.8) ^a^	0.7 (−1.8, 3.4) ^a,b^	1.0 (−2.4, 3.4) ^a,b^	−2.0 (−6.3, 1.8) ^b^	0.008
IL-10 (pg/mL)	0.2 (−0.3, 0.9) ^a^	0.1 (−0.3, 0.5) ^a,b^	0.0 (−0.6, 0.7) ^a,b^	−0.3 (−0.9, 0.2) ^b^	0.026
IL-8 (pg/mL)	0.0 (−1.1, 3.7)	−1.2 (−2.8, 0.0)	0.2 (−2.5, 2.4)	−0.5 (−2.2, 0.6)	0.378

Data are presented as mean ± standard deviation or median (25th and 75th percentile). AA = Placebo for 6 weeks; AB = Placebo for 6 weeks then SXRG84 treatment for 6 weeks; BB = SXRG84 treatment for 6 weeks; BA = SXRG84 treatment for 6 weeks then placebo for 6 weeks. Change determined by median post value (6 or 12 weeks) minus median baseline value; * *p*-value determined by ANCOVA using absolute data (adjusted means) from 6 weeks (for placebo and active group) and 12 weeks (for placebo then active group and active then placebo group) using baseline data as a covariate. Values with different superscript letters denote statistical significance (*p* < 0.05). For PASI data *n* = 25, 25, 19, 18, For CRP *n* = 25, 25, 18, 18, For inflammatory cytokine data *n* = 25, 25, 19, 18.

**Table 4:** Skin conditions that responded to SXRG84 intervention.

Skin Condition	Number That Participated in Trial	Responders, *n* (%)
Psoriasis	30	8 (27%)
Eczema	2	2 (100%)
Rosacea	2	0
Dermatitis	5	0
PPK	1	0
DSAP	1	0
PPP	3	0
Total	44	10 (23%)

PPK, palmar plantar keratoderma; DSAP, disseminated superficial actinic porokeratosis; PPP, palmar plantar psoriasis.

**Table 5:** ANCOVA analysis of responders vs. non-responders after SXRG84 treatment.

Outcome Measure	Responder vs. Non-Responder Mean Difference (Standard Error)	*p*-Value
PASI	−1.56 (0.87)	0.08
VAS	3.08 (0.76)	<0.001
DQLI	−2.04 (1.01)	0.049
C-reactive protein (mg/L)	−1.97 (2.26)	0.39
IFN-gamma (pg/mL)	−0.32 (0.76)	0.68
IL-1 beta (pg/mL)	−3.56 (2.68)	0.19
IL-6 (pg/mL)	−2.37 (2.36)	0.32
TNF-alpha (pg/mL)	−2.55 (1.63)	0.13
IL-10 (pg/mL)	−0.38 (0.27)	0.17
IL-8 (pg/mL)	−0.79 (1.66)	0.64

Data presented as mean difference (standard error).

**Table 6:** Significant Spearman correlations at baseline between skin measures and gut microbiota genera.

Skin Measure	Genus	Spearman’s Correlation Co-Efficient	*p*-Value
Baseline PASI score	*Peptoclostridium*	0.335	0.04
Baseline VAS	*Clostridium*	0.367	0.02
	*Coprobacter*	−0.480	0.03
	*Faecalibacterium*	0.322	0.04
	*Flavobacterium*	−0.434	0.04
	*Mogibacterium*	−0.604	0.00
	*Peptococcus*	−0.460	0.0
Baseline DQLI	*Dialister*	0.453	0.03
	*Lactobacillus*	0.426	0.03
	*Peptoclostridium*	0.372	0.02
	*Streptococcus*	−0.418	0.01

**Table 7:** Groups used for ANCOVA analysis.

AA: 6-week measure after placebo consumption
AB: 12-week measure after placebo (for 6 weeks) then SXRG84 treatment consumption (for 6 weeks)
BB: 6-week measure after SXRG84 treatment consumption
BA: 12-week measure after SXRG84 treatment (for 6 weeks) then placebo consumption (for 6 weeks)

## Data Availability

Data are not publicly available due to participant confidentiality as defined by the ethics committee. De-identified data are available on request from the corresponding author.

## Supplementary Materials

The following supporting information can be downloaded at: https://www.mdpi.com/article/10.3390/md21070379/s1, Figure S1: a, b, c. Participant with palmar plantar keratoderma (PPK) prior to SXRG84 treatment (a), during the trial after 3 weeks of SXRG treatment (b), and after the trial when treatment had ceased (c), Figure S2: a, b. Subject with psoriasis prior to SXRG treatment (a) and after six weeks of SXRG treatment (b).
