# Supplementary material for: Oral Supplementation with Algal Sulphated Polysaccharide in Subjects with Inflammatory Skin Conditions: A Randomised Double-Blind Placebo-Controlled Trial and Baseline Dietary Differences
Source: Mar Drugs. 2023 Jun 27;21(7):379. doi: 10.3390/md21070379 (PMC10381427; doi:10.3390/md21070379)
Supplement: Supplementary file 1 [file marinedrugs-21-00379-s001.zip › marinedrugs-2392349-supplementary.pdf]

Article Supplementary S1

Oral Supplementation with algal sulphated polysaccharide in subjects with inflammatory skin conditions: A randomised double-blind placebo-controlled trial and baseline dietary differences

Lauren A. Roach [1], Barbara J. Meyer [1,*], J. Helen Fitton[2] and Pia Winberg [3]

[1] School of Medical, Indigenous and Health Sciences, Molecular Horizons, Illawarra Health and Medical Research Institute, University of Wollongong, Wollongong, NSW 2522, Australia; lroach@uow.edu.au
[2] RDadvisor, Hobart, TAS 7006, Australia; drfitton@rdadvisor.com
[3] Venus Shell Systems Pty Ltd., Nowra, NSW 2540, Australia; pia@venusshellsystems.com.au
* Correspondence: bmeyer@uow.edu.au

Two skin conditions were examined as case studies and are presented here as supplementary material. The first case is Palmoplantar Keratoderma (PPK)which is characterised by hyperkeratosis of the palms and soles, a persistent thickening of the epidermis [1]. PPK can be characterised as either inherited or acquired. Hereditary PPK tends to present in childhood and its incidence can be traced throughout family histories [2]. Acquired PPK may result from exposure to certain chemicals, malnutrition, certain drugs, systemic diseases such as hypothyroidism, malignancy, and other inflammatory dermatoses such as psoriasis [2]. Traditional topical treatments include urea, salicylic acid, lactic acid, retinoids and corticosteroids [2]. Systemic treatment such as retinoid therapy may also be used [3].

The second skin condition case is of Psoriasis – a common immune-mediated skin disease [4] due to increased proliferation of keratinocytes. The most common form, psoriasis vulgaris is characterised by red, scaly and raised plaques of skin and pruritis is found in 70-80% of psoriasis patients [5]. The main etiology of psoriasis is a genetic predisposition and immunological disturbances however, stress, smoking, infections, and some drugs can also influence the condition. Psoriasis often begins in either late adolescence or early adulthood and then often remains for life [4, 5]. Treatments include systemic therapies, such as Infliximab and non-biologics such as methotrexate, phototherapies such as laser and UV treatments and topical corticosteroids [6]. Ingestion of foods that can shift the gut flora composition can also affect improvement in psoriatic type skin conditions [7] .

Case Reports

1. CASE REPORT #1

The subject was a 51 year old female suffering from PPK who was enrolled in a clinical trial (Biobelly 1 [8]). She was randomly assigned to the 4g treatment group of seaweed dietary fibre. At the beginning of the trial she presented with PPK on the palmar surface only, had no family history of this condition, and had been experiencing these symptoms chronically for two years. She required constant bandaging of her hands to help manage the pain and protect the epidermis on her palms.

She monitored her PPK throughout the six-week trial by taking photos of her hand. Figure S1 shows the participants hand prior to the trial (Figure S1a), during the trial after three weeks on the supplements, which is how it remained for the following three weeks of the trial (Figure S1b), and after the trial when the participant ceased the supplement (Figure S1c). As apparent in Figure S1b the PPK essentially cleared up whilst on SXRG84 and the subject was able to remove her bandages. After three

weeks of ceasing the capsules she had to revert to bandaging her hands again as the condition reverted. The participant had not been using any other treatments for the PPK. The participants blood parameters remained the same throughout the trial with the exception of a drop in fasting glucose (6.2 to 5.1mmol/L), the participants white cell count started and remained in the normal range. C-reactive protein levels remained unchanged throughout the trial with a level of 3 mg/L reported at baseline and post-intervention.

Since this dramatic result she has taken SXRG84 to test outside of the constraints of the clinical trial. The same effect was found twice more with the alleviation of symptoms whilst on the supplements and the symptoms returning when supplements were ceased. The dosage of 4g appeared effective each time.

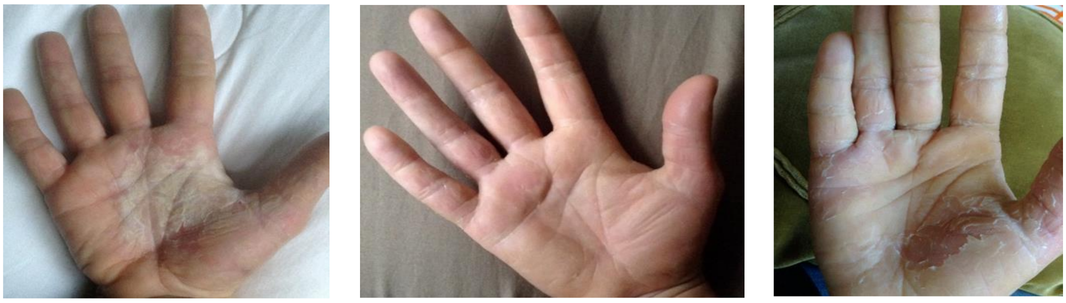

(**a**) (**b**) (**c**)

Figure S1. a, b, c. Participant with palmar plantar keratoderma (PPK) prior to SXRG84 treatment (a), during the trial after 3 weeks of SXRG treatment (b), and after the trial when treatment had ceased (c).

CASE REPORT #2

The subject was a 42 year old female who has been suffering from psoriasis for over ten years. She was provided with the seaweed dietary fibre extract but was not formally part of the clinical trial described above. She took photographs of her legs before commencing the supplements and after six weeks. Prior to beginning the supplements her legs appeared red and scaly (figure **S2**a). The subject consumed six capsules per day for six weeks, this was equivalent to 2g of the seaweed dietary fibre. After the six weeks the subjects legs were much improved (figure **S2**b). The subject reported no changes to diet or commencing any other psoriasis treatment.

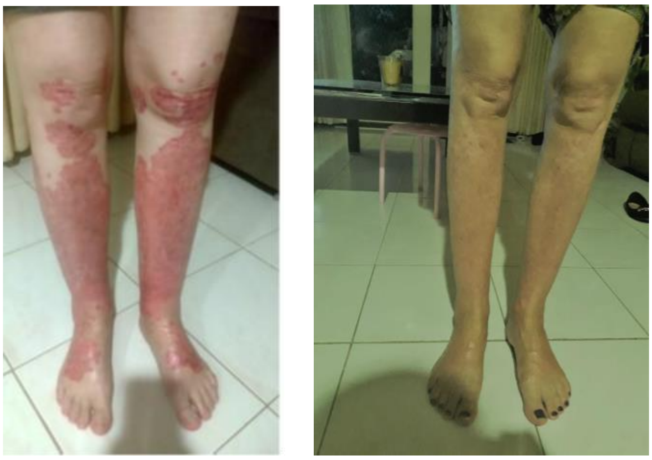

(**a**) (**b**)

Figure S2. a, b. Subject with psoriasis prior to SXRG treatment (a) and after six weeks of SXRG treatment (b).

To our knowledge these are the first case reports of dietary seaweed fibre ameliorating the symptoms of PPK and Psoriasis. Improvements in psoriasis have been linked to other dietary interventions, such

as energy restrictions with enriched omega-3 long chain polyunsaturated fatty acids [9] and gluten free diets [10]. Of interest from Case # 1 who suffered from PPK is the participants reduction in fasting plasma glucose after six weeks on the supplements. Commencing the trial, our participant was considered to be pre-diabetic based on fasting glucose results. Whereas at the end of the trial she was in an acceptable normal range for fasting glucose.

References

1. Guerra, L.; Castori, M.; Didona, B.; Castiglia, D.; Zambruno, G., Hereditary palmoplantar keratodermas. Part I. Non-syndromic palmoplantar keratodermas: classification, clinical and genetic features. *Journal of the European Academy of Dermatology and Venereology : JEADV* **2018,** *32* (5), 704-719.
2. Patel, S.; Zirwas, M.; English, J. C., 3rd, Acquired palmoplantar keratoderma. *American journal of clinical dermatology* **2007,** *8* (1), 1-11.
3. Schiller, S.; Seebode, C.; Hennies, H. C.; Giehl, K.; Emmert, S., Palmoplantar keratoderma (PPK): acquired and genetic causes of a not so rare disease. *Journal der Deutschen Dermatologischen Gesellschaft = Journal of the German Society of Dermatology : JDDG* **2014,** *12* (9), 781-8.
4. Lowes, M. A.; Bowcock, A. M.; Krueger, J. G., Pathogenesis and therapy of psoriasis. *Nature* **2007,** *445* (7130), 866-73.
5. Reich, A.; Szepietowski, J. C., Frontiers in Neuroscience Clinical Aspects of Itch: Psoriasis. In *Itch: Mechanisms and Treatment*, Carstens, E.; Akiyama, T., Eds. CRC Press/Taylor & Francis© 2014 by Taylor & Francis Group, LLC.: Boca Raton (FL), 2014.
6. Hamilton, M. P.; Ntais, D.; Griffiths, C. E.; Davies, L. M., Psoriasis treatment and management - a systematic review of full economic evaluations. *The British journal of dermatology* **2015,** *172* (3), 574-83.
7. Al-Ghazzewi, F. H.; Tester, R. F., Impact of prebiotics and probiotics on skin health. *Beneficial microbes* **2014,** *5* (2), 99-107.
8. Roach, L. A.; Meyer, B. J.; Fitton, J. H.; Winberg, P., Improved Plasma Lipids, Anti-Inflammatory Activity, and Microbiome Shifts in Overweight Participants: Two Clinical Studies on Oral Supplementation with Algal Sulfated Polysaccharide. *Marine Drugs* **2022,** *20* (8), 500.
9. Guida, B.; Napoleone, A.; Trio, R.; Nastasi, A.; Balato, N.; Laccetti, R.; Cataldi, M., Energy-restricted, n-3 polyunsaturated fatty acids-rich diet improves the clinical response to immuno-modulating drugs in obese patients with plaque-type psoriasis: a randomized control clinical trial. *Clinical nutrition (Edinburgh, Scotland)* **2014,** *33* (3), 399-405.
10. Wolters, M., Diet and psoriasis: experimental data and clinical evidence. *The British journal of dermatology* **2005,** *153* (4), 706-14.
